# Prognostic value of tumor deposits and their different response to neoadjuvant therapy in locally advanced rectal cancer

**DOI:** 10.1371/journal.pone.0340000

**Published:** 2026-01-13

**Authors:** Di Zhou, Junjun Zhou, Bin Hu, Yinjue Yu, Yuhai Bian, Cuiping Yang, Yongrui Bai, Haiyan Chen

**Affiliations:** 1 Department of Radiation Oncology, Ren Ji Hospital, Shanghai Jiao Tong University School of Medicine, Shanghai, China; 2 Department of Gastrointestinal Surgery, Ren Ji Hospital, Shanghai Jiao Tong University School of Medicine, Shanghai, China; Universita Cattolica del Sacro Cuore, ITALY

## Abstract

**Background:**

Tumor deposits (TDs) may have a worse prognosis in rectal cancer, but their significance in the neoadjuvant era is less certain. Post-treatment TDs might even be a sign of tumor response. The present study aimed to assess the clinical significance of TDs detected before and after neoadjuvant therapy, and to investigate the impact of neoadjuvant therapy-induced TDs changes on oncological outcomes.

**Methods:**

A retrospective cohort analysis using our hospital records from 2017 to 2019 was carried out. All patients received preoperative long-course chemoradiotherapy and part of them received total neoadjuvant therapy.

**Results:**

A total of 132 patients with cT_3-4_N_+_M_0_ were included. mrTDs were observed in 40 (30.3%) patients. 40% of the patients had two or more mrTDs. 64.4% of mrTDs located in the mesorectal fat. mrTDs were associated with mrT4 stage, lymph node invasion, threatened mrMRF, and positive mrEMVI. 51.4% of mrTDs positive patients achieved complete response after neoadjuvant therapy. 3‐year disease‐free survival (DFS) and overall survival (OS) were worse in mrTD positive patients (3y-DFS: 42.5% vs 73.9%, *P* < 0.001; 3y-OS: 55% vs 82.9%, *P* < 0.001). Among the patients with mrTDs, those who became ypTDs- after neoadjuvant therapy had better outcome compared to the ypTDs+ patients (3y-DFS: 52.6% vs 22.2%, *P* = 0.022; 3y-DMFS: 63.2% vs 27.8%, *P* = 0.025). Distant metastasis occurred earlier and more frequently in ypTDs+ group, and multiple metastasis were more common. ypTDs and TDs’ different response to neoadjuvant therapy were prognostic factors of overall survival in multivariate analysis.

**Conclusions:**

The presence of mrTDs and the poor regression of mrTDs in cT_3-4_N_+_M_0_ rectal cancer after neoadjuvant treatment are associated with advanced disease and worse outcome. Patients with ypTDs+ after neoadjuvant therapy have dismal outcome, which call for more innovative treatment.

## Introduction

The treatment of locally advanced rectal cancer (LARC) is more and more diversified and personalized. Total neoadjuvant therapy (TNT) as an alternative treatment for LARC is now supported by the National Comprehensive Cancer Network. Both ESMO and ASCO recommend high-resolution magnetic resonance imaging (MRI) for the evaluation of LARC [1,2]. Based on the distance from the anal margin, T stage, N stage, mesorectal fascia (MRF) and extramural venous invasion (EMVI), different neoadjuvant therapy modalities are recommended for patients at different risk of locoregional recurrence.

Tumor deposits are defined as discrete tumor nodules within the lymphatic drainage area of the primary tumor. These nodules are discontinuous from the main tumor mass and lack evidence of lymph node tissue. Furthermore, they do not contain identifiable vascular or neural structures. Typically, they are located in the subserosa, mesentery, or the non-peritonealized pericolonic or perirectal adipose tissue. Pathologically, metastatic lymph nodes typically exhibit a rounded contour, a capsular structure, and may contain residual lymphoid follicles. In contrast, tumor deposits present as irregularly shaped, poorly demarcated masses of tumor cells devoid of these characteristic nodal architectural features. Radiologically, tumor deposits also demonstrate distinguishing characteristics, such as irregular morphology, indistinct margins, and the presence of lobulation or spiculation. Their attenuation on non-contrast scans is often similar to that of the primary tumor, and they typically show marked enhancement post-contrast administration. These imaging features are instrumental in differentiating them from the generally well-defined, regularly shaped metastatic lymph nodes.

The presence of tumor deposits (TDs) was associated with significantly poorer survival outcome of colorectal cancer. The CALGB/SWOG 80702 study and the IDEA study suggest that pathologic TDs are an independent negative prognostic factor, with a linear relationship between disease‐free survival (DFS), overall survival (OS) and the number of tumor deposits. More than a quarter of patients with stage III colon cancer are TDs positive [3]. A national study cohort in which 3769 patients were analyzed suggests that TDs have a negative impact on prognosis in rectal cancer. In TDs positive patients, local recurrence and distant metastasis rates at 5 years were 6.3% [95% CI 3.8–8.8%] and 38.9% [95% CI, 33.6–43.5%] compared to 2.7% [95% CI, 2.1–3.3%] and 14.3% [95% CI, 13.1–15.5%] in TDs negative patients. In multivariable regression analysis, risk of local recurrence and distant metastasis were increased. Overall survival at 5 years was 68.8% [95% CI, 64.4–73.4%] in TDs positive patients and 80.7% [95% CI, 79.4–82.1%] in TDs negative patients. Thus, efforts should be made to diagnose TDs positive rectal cancer patients preoperatively [4].

The above data were mainly derived from upfront surgery when it turns to the neoadjuvant era, the significance of TDs become less clear. With the widespread application of high-resolution MRI and functional imaging, TDs have recently been noted to be visible on MRI. It is possible for us to distinguish TDs from lymph node metastases with MRI and TDs are predictors for diminished OS and DFS.

In the current study, we evaluated the incidence and features of mrTDs and the response to neoadjuvant chemoradiotherapy (CRT) to investigate the prognostic effect of TDs and their association with clinical outcomes.

## Materials and methods

### Patients and pretreatment evaluation

A total of 136 patients with cT_3-4_N + M_0_ rectal cancer were treated with CRT followed by surgery at our hospital from 15/08/2017–19/07/2019. From this cohort, only one patient was excluded for sudden cardiac death during CRT and three patients were lost to follow-up. The clinical and imaging characteristics, treatment, and outcomes of the remaining 132 patients were retrospectively analyzed.

This study was approved by the Shanghai Jiao Tong University School of Medicine, Ren Ji Hospital Ethics Committee [2017−053]. The requirement for individual informed consent was waived by the Shanghai Jiao Tong University School of Medicine, Ren Ji Hospital Ethics Committee because of the retrospective nature of the study.

### Preoperative evaluation

All patients underwent evaluation including digital rectal examination, colonoscopy with biopsy, magnetic resonance imaging (MRI) and/or transrectal ultrasound or abdominal computerized tomography (CT), chest CT and/or positron emission tomography (PET)-CT when necessary. When MRI shows that the distance between the tumor, lymph nodes, and the tumor signal of EMVI and MRF is less than 1 millimeter, it can be diagnosed as threatened mrMRF. We adopt high-resolution T2-weighted imaging (T2WI), incorporated multi-b-value diffusion-weighted imaging (DWI), and implemented joint diagnosis by at least two radiologists specialized in a blinded analysis.

### Treatment

All of the patients (n = 132) were treated with preoperative long-course CRT followed by surgery. Three-dimensional conformal radiotherapy or intensity-modulated radiotherapy was administered; patients received 45Gy to the pelvis in 25 fractions at 1.8Gy per fraction, with a 5.4–9Gy boost to the tumor. Capecitabine (825 mg/m^2^ bid) was administered during radiotherapy. Neoadjuvant chemotherapy was administered for a total of 12 weeks preoperatively, and adjuvant CAPOX was administered for 12 weeks postoperatively. We usually recommend total neoadjuvant therapy (TNT) for patients who are at high risk of recurrence, such as those with T4 stage, positive MRF, positive EMVI, involvement of the levator ani muscle, N2 stage, or lateral lymph node metastasis.

### Follow-up

Patients were followed up every 3 months during the first 2 years, and thereafter every 6–12 months. At each follow-up patients underwent physical examination, serum carcinoembryonic antigen level measurement, chest radiography, and abdominal and pelvic CT or MRI. Bone scintigraphy and colonoscopy were performed annually. Authors didn’t have access to information that could identify individual participants during or after data collection.

### Response evaluation

cCR was defined as the absence of clinically detectable residual primary tumor on clinical examination and endoscopy at least 8 weeks after completion of radiotherapy. Pathological complete response (pCR) was defined as absence of residual cancer cells in the primary tumor site and in regional lymph nodes at pathology after radical surgery. Local recurrence (LR) was defined as any recurrence in the pelvic radiation field. Distant metastasis (DM) was defined as recurrence outside the radiation field. Disease free survival (DFS) was defined as the time interval from the start of CRT to the recurrence of the disease, progression on neoadjuvant chemoradiation resulting in inoperability, or death from any cause. Distant metastasis–free survival (DMFS) was defined as the duration from the start of CRT to the time of diagnosis of distant metastasis. Overall survival (OS) was defined as the duration from the start of CRT to the time of death or to end of the follow-up period.

### Statistical analysis

Survival curves were generated using the Kaplan–Meier method, and the differences between the curves were analyzed by the log-rank test. The Cox proportional hazards model was used for the multivariate assessment of the predictors of overall survival, and these results are presented as hazard ratios and 95% confidence intervals. The validity of the Cox model relies on the proportional hazards (PH) assumption. This was assessed using the Schoenfeld residual test, where a p-value >0.05 indicates that the variable satisfies the PH assumption. For any variables found to violate this assumption, they are explicitly addressed in the Results section and were considered for inclusion in the model as stratification variables or by incorporating time-dependent covariates. Prior to constructing the multivariable Cox regression model, variance inflation factor (VIF) analysis was performed on all continuous variables to assess multicollinearity. A VIF value below 10 was considered indicative of no substantial multicollinearity. All statistical tests were two-sided, and statistical significance was at *P* ≤ 0.05. SPSS for Windows, version 23.0 (IBM Inc., Armonk, NY, USA) was used for data analysis.

## Results

Overall, 132 patients were included in the analysis, with cT3 (35.6%, 47/132) and cT4 (64.4%, 85/132) rectal cancers. 75.8% (100/132) of the patients were diagnosed with lymph node metastasis. Lower rectal cancer, distance from anal average is less than 5 cm, accounted for 51.5% (68/132). 109 patients received induction/consolidation chemotherapy with modified FOLFOX-6 or CAPEOX two to four cycles before/after long-course CRT, while the other 23 patients only had long-course CRT with concomitant oral fluoropyrimidine. 32 patients received induction chemotherapy, while 77 patients received consolidation chemotherapy. Eight patients who achieved clinical complete response (cCR) chose to watch and wait. Three patients with clinical partial response (cPR) refused radical surgery and another six patients remained clinical stable disease (cSD) after neoadjuvant therapy. Then 115 patients received total mesorectal excision (TME) (include abdominal perineal resection and low anterior resection) and at least four cycles of adjuvant chemotherapy (S1 Fig).

The median follow-up time was 33months (5–87 months). For the whole cohort, 3- year OS and DFS were 61% and 55% respectively. 14.4% of the patients had local recurrence and 32.5% developed distant metastasis.

### Baseline mrTDs status

mrTDs were detected in 40 of 132 patients (30.3%) before treatment. mrTDs were associated with advanced mrT stage, lymph node metastasis (especially pelvic lymph nodes metastasis), threatened mrMRF and positive mrEMVI (*P* < 0.05, Table 1). The occurrence of mrTDs was more correlated with mrEMVI + (correlation coefficient 0.588, *P* < 0.001).

**Table 1:** The relationship between tumor deposits and pretreatment clinical characteristics.

	Patients Without mrTDs (n = 92)		Patients With mrTDs (n = 40)		*P*
	n	%	n	%	
Age, years					
<65	59	64.1	26	65.0	1.0
≥65	33	35.9	14	35.0	
Sex					
Male	74	80.4	30	75.0	.494
Female	18	19.6	10	25.0	
Clinical T stage					
T_3_	38	41.3	9	22.5	**.048**
T_4_	54	58.7	31	77.5	
Clinical N stage					
N_0_	29	31.5	3	7.5	**.003**
N_+_	63	68.5	37	92.5	
mrMRF					
MRF-	30	32.6	4	10.0	**.009**
MRF+	62	67.4	36	90.0	
mrEMVI					
EMVI-	68	73.9	8	20.0	**<0.001**
EMVI+	24	26.1	32	80.0	
mrPLN					
PLN-	84	91.3	29	72.5	**.007**
PLN+	8	8.7	11	27.5	
Distance from anal verge					
Low (0–5 cm)	47	51.1	21	52.5	1.0
Mid (5–10 cm)	45	48.9	19	47.5	

The prefix “mr” denotes MRI-detected features. TDs: tumor deposits; MRF: mesorectal fascia; EMVI: extramural venous invasion; PLN: pelvic lymph node.

**Fig 1:**
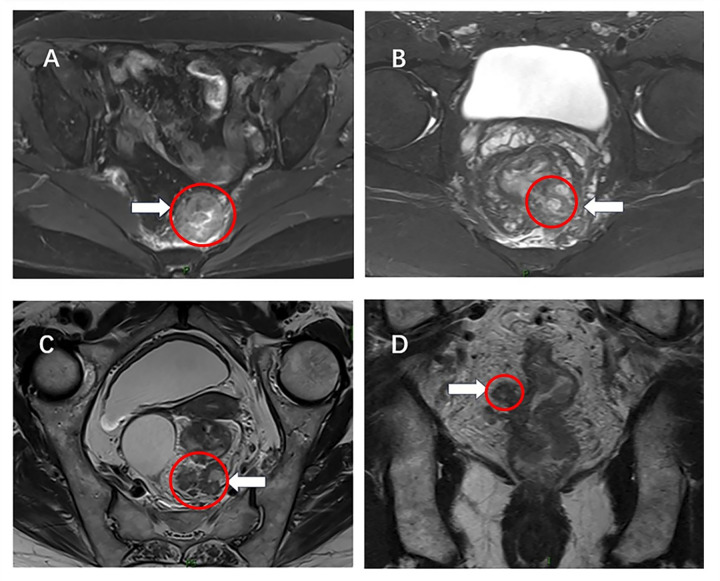
Axial T2-weighted image. (A) demonstrating tumor deposit (white arrow) with irregular shape, signal heterogeneity and is seen invading the anterior sacral fascia. (rectal cancer has not been shown in the image). (B) demonstrating tumor deposit (white arrow) with irregular shape, signal heterogeneity and irregular margins and is seen invading the mesorsstal fascia. (C) from another patient depicting multiple tumor deposits (white arrow) which are close to the mesorsstal fascia. Coronal T2-weighted image (D) from another patient depicting a tumor deposit (white arrow) with subcircular shape and smooth margins.

The total number of mrTDs was 73 in the whole group. 40% (16/40) of the patients had two or more mrTDs. mrTDs occurred in the mesorectal fat (64.4%, 47/73), the anterior sacral area (30.1%, 22/73) and the pelvic wall area (5.5%, 4/73). 20% patients (8/40) had mrTDs in synchronous two areas. Nearly half of the mrTDs invaded adjacent organs or adhered tightly to the pelvic wall, fascia and muscles, which we called invasive mrTDs (Fig 1). The diameter of the nodules was 8–50 mm, with a mean diameter of 21.63 mm.

### Postoperative TDs status (ypTDs)

Among the 40 patients with positive mrTDs, 37 patients underwent surgery. Prior to surgery, the dissection scope and key focus areas of the operation were determined based on the regions of tumor deposits identified in the initial diagnosis, aiming to minimize omissions during surgery and pathological examination. The R0 resection rate was 86.5% (32/37) while the non-R0 resection rate was 13.5% (5/37). One case with non-invasive mrTDs was evaluated as cCR after neoadjuvant therapy and just underwent transanal endoscopic microsurgery (TEM). The other 2 cases were invasive mrTDs patients with poor regression after neoadjuvant therapy, and thus no chance for further operation. As shown in Table 2, R0 resection could be performed on all non-invasive mrTDs, but the R0 resection rate was lower for invasive mrTDs (*P* = 0.027; Table 2).

**Table 2:** Analysis of surgical resection of TDs after neoadjuvant therapy (n = 40).

Invasive mrTDs	Resection of TDs		
	R0	Non-R0	No surgery
–	20	0	1^*^
+	12	5	2^**^
Total	32	5	3

* The patient was evaluated as cCR after neoadjuvant therapy and just underwent a local resection. ** Two patients with poor regression and they didn’t take operation.

### Changes in TDs

**Fig 2:**
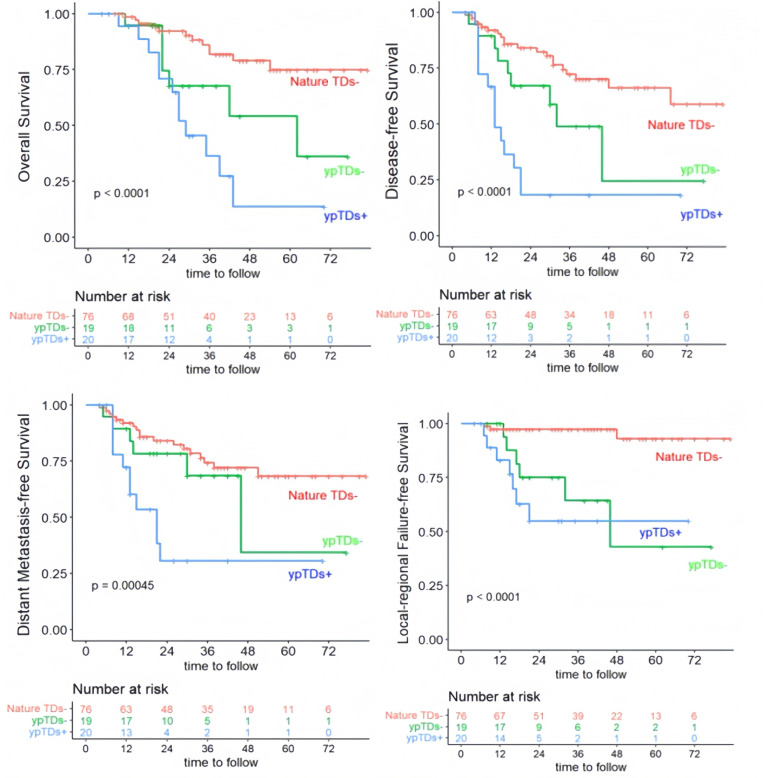
Survival Analysis according to the changes in TDs after neoadjuvant therapy. (A) overall survival, (B) disease-free survival, (C) distant metastasis-free survival and (D) local-regional failure-free survival.

After neoadjuvant therapy, 51.4% (19/37) of mrTDs positive patients achieved complete regression or became fibrosis (Details in S1 Table). This statement is in reference to complete regression of the TDs, not the complete regression of primary tumor. According to the changes of TDs patients were divided into three groups: nature TDs- group (n = 76) in which both mrTDs and ypTDs were negative, ypTDs- group (n = 19) in which mrTDs achieved complete regression after neoadjuvant therapy, and ypTDs+ group (n = 18). Notably, two patients with mrTD- exhibited pathological tumor deposits (ypTD+) after neoadjuvant therapy. After a median follow-up of 33months (range, 5–87months), 19 patients had died, with 7 in ypTDs- group and 12 in ypTDs+ group. 3‐year disease‐free survival (DFS) and overall survival (OS) were worse in mrTD positive patients (3y-DFS: 42.5% vs 73.9%, P < 0.001; 3y-OS: 55% vs 82.9%, P < 0.001). Among the patients with mrTDs, those who became ypTDs- after neoadjuvant therapy had better outcome compared to the ypTDs+ patients (3y-DFS: 52.6% vs 22.2%, P = 0.022; 3y-DMFS: 63.2% vs 27.8%, P = 0.025; Fig 2). Through univariate and multivariate analysis of OS, EMVI, PLN, ypTDs, ypN and changes in TDs after CRT were independent prognostic factors (Details in Table 3).

**Table 3:** Univariate and multivariate analysis of OS in LARC who received neoadjuvant chemoradiotherapy (n = 115).

Variables		Group	Patients Numbers	Univariate analysis		Multivariate analysis		
				95% CI	*P*	OR	95% CI	*P*
Patient characteristics	Sex	Male	90		0.577			
		Female	25					
	Age	< 65	78		0.706			
		≥65	37					
	Distance from anal verge	Low (<5 cm)	59		0.907			
		Upper/mid	56					
Pre-CRT MR	MRF	Negative	26		**0.009**			0.713
		Positive	89					
	EMVI	Negative	66	23.92-54.09	**0.000**	5.163	1.15-23.13	**0.032**
		Positive	49					
	PLN	Negative	98	18.75-39.26	**0.000**	4.177	1.62-10.77	**0.003**
		Positive	17					
	TDs	Negative	78	23.46-54.54	**0.000**			
		Positive	37					
Postoperative pathology	ypEMVI	Negative	90	21.98-56.02	**0.039**			0.344
		Positive	25					
	Margin involvement	Negative	108	21.57-32.43	**0.000**			0.573
		Positive	7					
	ypTDs	Negative	95	20.49-37.51	**0.000**	3578.523	28.18-454424.63	**0.001**
		Positive	20					
	ypT stage	T0	19		**0.01**			0.175
		T1-2	37					
		T3-4	59					
	ypN stage	N0	77		**0.000**	3.178	1.45-6.95	**0.004**
		N1	26					
		N2	12					
	Changes in TDs after CRT	Nature TDs-	78		**0.000**			**0.006**
		ypTDs-	20					
		ypTDs+	17					

The prefix “yp” denotes post-surgery pathologic features. TDs: tumor deposits; MRF: mesorectal fascia; EMVI: extramural venous invasion; PLN: pelvic lymph node.

The Kaplan-Meier curves for local recurrence-free survival(LRFS) displayed crossing trajectories, and the Schoenfeld residual test confirmed a violation of the proportional hazards assumption. Accordingly, we employed a Cox model with time-dependent covariates to accurately capture the dynamic nature of the hazard ratios, as illustrated in the time-varying hazard ratio plot. The analysis revealed distinct risk patterns for the different TD groups: ypTD- vs. nature TD-: The hazard relationship reversed over time. ypTD- was associated with a lower risk of recurrence in the early period (LRFS<20 months), but its risk increased sharply thereafter (LRFS>20 months), with the hazard difference widening over time. ypTD + vs. nature TD-: This group demonstrated a consistently higher risk compared to nature TD-, although the hazard ratio increased only gradually, showing no evidence of a similar risk reversal. These findings suggest distinct clinical implications: patients in ypTD-, while having a lower early recurrence risk, face a substantially elevated long-term risk, necessitating vigilant long-term follow-up. In contrast, patients in ypTD+ exhibit a persistently higher risk from the outset, albeit with a more stable trajectory, which may warrant consideration for more intensive early intervention.

### Recurrence pattern

During the follow-up, 9 patients (47.4%) in ypTDs- group versus 15 patients (75%) in ypTDs+ group experienced recurrences (*P* = 0.022). In ypTDs- group, 2 patients (10.5%) had locoregional recurrence (LR), 3 (15.8%) had distant metastasis (DM), and 4 (21.1%) had combined LR and DM. In ypTDs+ group, 2 patients (10%) had LR, 6 (30%) had DM, and 7 (35%) had combined LR and DM. Statistical analysis revealed a significant reduction in local recurrence rates in ypTDs- patients, with anastomotic recurrence being the most common pattern, whereas ypTDs+ patients exhibited a higher proportion of lymph node metastasis in the TDs region, indicating distinct recurrence mechanisms between the two groups. The ypTDs- group demonstrated a significantly lower distant failure rate (36.9% versus 65%, *P* = 0.025) than ypTDs+ group (S2 Fig).

Recurrence timing and frequency in accordance with the change of TDs in LARC patients are shown in Table 4. In the ypTDs+ group, 100% of the local recurrence and distant metastasis occurred in the first 2 years. In the ypTDs- group, 66.7% (4/6) of the local recurrence and 71.4% (5/7) of the distant metastasis occurred in the first 2 years after resection. In total, 28.6% (2/7) of distant metastasis occurred in the 3th to 4th year after surgery in the ypTDs- group. In the nature TDs- group, 50% (2/4) of the local recurrence and 66.7% (12/18) of the distant metastasis occurred in the first 2 years after resection and 33.3% (6/18) of distant metastasis still occurred in the 3th to 4th year after surgery.

**Table 4:** Recurrence pattern in LARC patients who received neoadjuvant therapy according to TDs status.

	Local Recurrence			Distant Metastasis		
	Nature TDs- (n = 4)	ypTDs- (n = 6)	ypTDs+ (n = 9)	Nature TDs- (n = 18)	ypTDs (n = 7)	ypTDs+ (n = 13)
Time after surgery (months)	No. of LR (%)	No. of LR (%)	No. of LR (%)	No. of DM (%)	No. of DM (%)	No. of DM (%)
≤12 months	2 (50%)	3 (50.0%)	5 (55.6%)	9 (50.0%)	4 (57.1%)	10 (76.9%)
12-24 months	0 (0%)	1 (16.7%)	4 (44.4%)	3 (16.7%)	1 (14.3%)	3 (23.1%)
24-36 months	1 (25%)	1 (16.7%)		5 (27.8%)	1 (14.3%)	
36-48 months	1 (25%)	1 (16.7%)		1 (5.5%)	1 (14.3%)	

Further analysis of distant metastasis sites according to ypTDs state in patients with mrTDs + was performed. The common sites of metastasis were liver, lung, bone, peritoneum, and extra-regional lymph nodes. Most of metastasis are synchronous multi-sites. In the ypTDs- group, 28.6% (2/7) of the distant metastasis are oligo-metastasis. One patient developed para-aortic lymph node metastasis and the other had a solitary lung metastasis. In the ypTDs+ group, 15.4% (2/13) of the distant metastasis are oligo-metastasis. One patient developed para-aortic lymph node metastasis and the other had a solitary liver metastasis. In the nature TDs- group, 33.3% (6/18) of the distant metastasis are oligo-metastasis. Four of them were solitary liver metastasis and the other two were solitary lung metastasis.

## Discussion

Definitions of what constitutes tumor deposits in colorectal cancer vary between different stage system [5–9]. Since 1935 when TDs were proposed by Gabriel for the first time, the definition of TDs has changed continuously. Growing evidence suggests TDs and lymph nodes have distinctly different prognostic implications. A post hoc analysis of the IDEA trial showed the presence of TDs was an independent prognostic factor for DFS in patients with stage III colon cancer. Of 1,454 pN1-staged patients, 35 (2.4%) were restaged as pN2 by the addition of TDs to the LNM count. These patients had a lower 3-year DFS rate than patients with tumors remaining as pN1 despite the addition of TDs to the LNM count (79.34% v 60.72%, respectively; *P* = 0.0151). Importantly, Patients restaged as having pN2 disease had similar DFS as patients initially classified as pN_2_. Adding the number of tumor deposits to the count of lymph node metastases improves the prognostication accuracy [10]. Another study also found TDs had a metastatic risk comparable to a pN_2_ stage. TDs were associated with a worse 3‐year DFS among pN_0_ (51.2% vs 79.8%; *P* < 0.001); pN_1_ patients (35.2% vs 70.1%; *P* = 0.004) but not among pN_2_ patients (37.5% vs 44.7%; *P* = 0.499) [11]. Similarly, the Swedish national Cohort study confirmed that TDs in rectal cancer were associated with increased recurrence risk and decreased survival. pN_1c_ patients had similar outcomes regarding local recurrence, distant metastasis and survival as pN_1a-b_ stage patients. TD-positive pN_1a-b_ patients had significantly worse outcomes while TDs did not affect outcomes in pN_2a-b_ patients [4].

In recent years, attention has been drawn to distinguish TDs from lymph node metastases on MRI. Lesion shape on MRI can be a useful predictor for distinguishing TDs from positive LNs in rectal cancer patients, with sensitivity and specificity of 90% and 68% respectively. The presence of irregular margins, heterogenous T2 signal intensity of the lesion and irregular shape were strongly associated with TDs. When interpreted along with MR texture parameter of skewness, accuracy is further improved [12]. More and more clinical centers are using T2WI-based multiregional radiomics and/or mono-exponential, bi-exponential, and stretched-exponential models of multi-b-value diffusion-weighted imaging (DWI) to predict TDs in patients with rectal cancer [13,14].

We recorded the presence of MRI features of TDs suggesting a potential origin to help improve our understanding of tumor spread in the future. Firstly, some TDs may act as satellites of the main tumor to increase the field of invasion. Others are isolated nodules that are distant from the primary tumor. The diagnosis of invasive mrTDs helps us to predict the possibility of resection of TDs. Secondly, TDs are mainly distributed in the lymphatic drainage pathways of rectal cancer, such as the mesorectal region, the anterior sacral region, and the obturator region. TDs may be the manifestation of lymph node metastasis in different periods of rectal cancer. Certain groups suggest that TDs could be the complete replacement of an LN by metastatic tumor, whereas others consider TDs as in-transit metastases, where tumor cells spread through lymphatic channels and form tumors before reaching LNs [15]. Thirdly, mrTDs were strongly associated with increased mrT stage, lymph node metastasis (especially pelvic lymph nodes metastasis), threatened mrMRF and positive mrEMVI in the current study. We detected there was a significant association between mrTDs and mrEMVI. The relationship between pEMVI and pTDs was demonstrated in a meta-analysis, in which eight studies demonstrated that pTDs occurred two-times more frequently in pEMVI positive patients [16,17]. It suggested that a proportion of TDs may represent blood-borne spread and that TDs were early form of metastatic disease in rectal cancer. The recurrence pattern of mrTDs+ patients shown below is also consistent with this hypothesis.

In the present study, we retrospectively analyzed 132 LARC patients after neoadjuvant therapy to evaluate the prognostic significance of TDs, both mrTDs and ypTDs, and reported the pattern of recurrence after surgery in those mrTDs+ patients for the first time. Previous meta-analyses have shown TDs were independently associated with a poor prognosis [17,18]. There was a pooled hazard ratio for 5-year DFS was 2.3 (95% confidence interval [CI]: 1.8 to 2.9), and that for overall survival was 2.5 (95% CI: 1.9 to 3.3) in ypTDs+ patients after chemoradiotherapy. In contrast, a recent study indicated that the groups with mrEMVI/mrTDs had poorer outcomes than those without and this was irrespective of pathology nodal status. On multivariable analysis of MRI-based prognostic factors, only mrTDs and mrEMVI remained a significant adverse prognostic marker for both OS and DFS as well as for overall recurrence and distant recurrence [19]. Similarly, we revealed worse OS, DFS, DMFS and LRFS in either mrTDs+ or ypTDs+ patients.

The ESMO consensus and ASTRO guidelines for LARC suggest that patients should be classified according to clinical stage TNM, involvement of MRF, EMVI, size, level and localization [1,2]. Other factors, such as cN stage, vascular and nerve invasion are also relevant [20,21]. Preoperative treatment modalities are different in patients with different risk stratification. Chinese Society of Clinical Oncology Colorectal Cancer Guidelines 2023 (CSCO 2023) added TDs and recommended radiologists labeling. It coincides with our study that mrTDs are associated with survival. We can obtain the characteristics of TDs, such as the size, number, location and morphology, before neoadjuvant therapy by MRI, as opposed to pathology where only a snapshot of the mesorectum, both post-treatment and ex-vivo, is available. The status of TDs before neoadjuvant therapy supplemented the risk stratification factors, then the mode and the intensity of neoadjuvant therapy will be adjusted. In addition, we should pay more attention to dissecting the regions where mrTDs are located to reduce the risk of R1 resection during the TME surgery.

However, some changes will occur after neoadjuvant therapy, such as decreased number of TDs, degenerative tumor cells, the formation of mucous lakes, and tissue fibrosis. These changes may influence the accuracy of the prognostic and staging values of TDs in patients with neoadjuvant therapy. In mrTDs positive patients, we found that patients with good regression (ypTDs- group) to TNT had better DFS and DMFS than those with poor regression (ypTDs+ group). 3-year DFS were 52.6% in ypTDs- group and 22.2% in ypTDs+ group (*P* = 0.022), while 3-year DMFS were 63.2% and 27.8% in two groups (*P* = 0.025) respectively. Although there was no statistically significant difference in OS, there may be a trend of survival benefit with the extension of follow-up time. Our study indicated that the ypTDs+ group was associated with significantly higher distant recurrence risk than the ypTDs- group in mrTDs+ LARC. Moreover, we found that recurrence occurred not only more frequently but also earlier in the ypTDs+ group than those in the ypTDs- group. 77.8% of patients in ypTDs+ group was suffered from recurrence within 2 years after surgery. Distant metastasis was the main failure mode, in which multiple metastasis were more common. Based on the significant association between ypTDs+ status and early recurrence (within 2 years), intensive surveillance (e.g., quarterly imaging, tumor marker assessments and ctDNA [22]) is strongly recommended for this subgroup. In contrast, patients with ypTDs- or nature TDs- should receive extended follow-up until the 4th postoperative year, given their residual risk of late distant metastasis.

Individualized treatment plans and surveillance strategies could be developed for mrTDs+ in LARC. In those patients after neoadjuvant therapy, the finding of persistent TDs was a marker of particularly poor prognosis. Those who showed a good treatment response and became ypTDs- had better outcomes. TNT may be a highly effective treatment for this group of patients, the true high-risk group of patients who are likely to see the most benefit with the use of TNT. Meanwhile, the regression of TDs after neoadjuvant treatment may provide a basis for subsequent adjuvant therapy. If TDs are located in the pelvic wall, an extended lateral dissection is required (not usually considered in Western countries). It is more difficult for invasive mrTDs to achieve R0 resection so that it increases the risk of local recurrence and the tumor may propagate through a network of intra fascial capillaries leading to distant failure. We need to consider whether it is necessary to boost local dose in preoperative radiotherapy and combine target drugs in the neoadjuvant therapy. Furthermore, a more frequent surveillance within the first 2 years should be recommended for ypTDs+ patients, and follow-up every 3 months for ypTDs+ patients may be beneficial. More individualized stratification of recurrence risk should be developed based on multicenter data in future.

Our study aimed to evaluate the significance of the presence of TDs before and after neoadjuvant therapy, as well as the changes of them induced by neoadjuvant therapy regarding oncological outcomes. It is limited by the retrospective nature and small sample size. The absence of data on the effect of TD counts on prognosis, heterogenous neoadjuvant treatment strategies, short follow-up are also limitations. Subsequently, expanding the sample size and refining the stratification based on the number of TD will help us verify whether the risk of recurrence and metastasis increases in a stepwise manner with the increase in the number of TDs. There are still many questions about why certain patients demonstrate good regression in response to neoadjuvant treatment and others do not. Regression of TDs certainly appears to be prognostically significant, but it is not clear whether this is simply a function of ‘better biology’ at baseline, or whether it is the induction of regression itself that mediates the improved outcomes. More researches in this area are needed, including in prospectively recruited trials, in order to answer these questions. If TDs regression truly lead to improved survival, it would be of great interest to know if induction chemotherapy or total neoadjuvant therapy combined with immunotherapy would be more effective.

## Conclusions

In locally advanced rectal cancer, mrTDs and the poor regression of mrTDs after neoadjuvant therapy are negative prognostic factors of OS. Recurrence patterns is different according to the response of TDs to CRT. We suggested more attention should be paid to the response of TDs after neoadjuvant treatment, considering their prognostic significance.

## Supporting information

S1 Fig Flowchart of included rectal cancer patients for analysis. (TIFF)

S2 Fig Recurrence patterns in nature TDs-, ypTDs- and ypTDs+ subgroups. (LR, locoregional recurrence; DM, distant metastasis). (TIFF)

S1 Table Analysis of the relationship between mrTDs and ypTDs (n = 115). (DOCX)

S1 File Baseline characteristics of enrolled patients with locally advanced rectal cancer (n = 132). (XLSX)
