# Supplementary figures and images for: Prognostic value of tumor deposits and their different response to neoadjuvant therapy in locally advanced rectal cancer
Source: PLoS One. 2026 Jan 13;21(1):e0340000. doi: 10.1371/journal.pone.0340000 (PMC12798964; doi:10.1371/journal.pone.0340000)

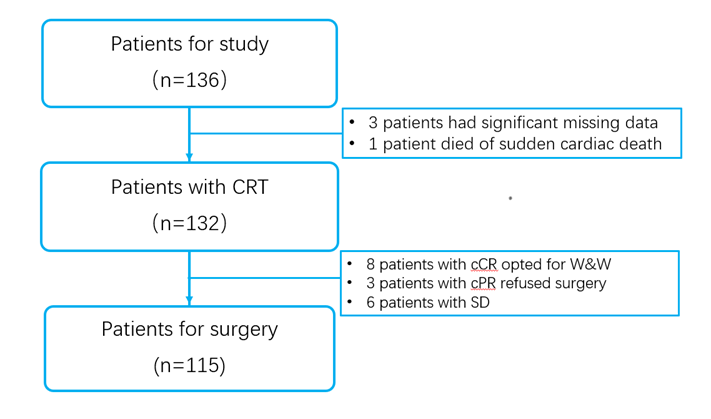

Supplement: S1 Fig — (TIFF) [file pone.0340000.s001.tiff]

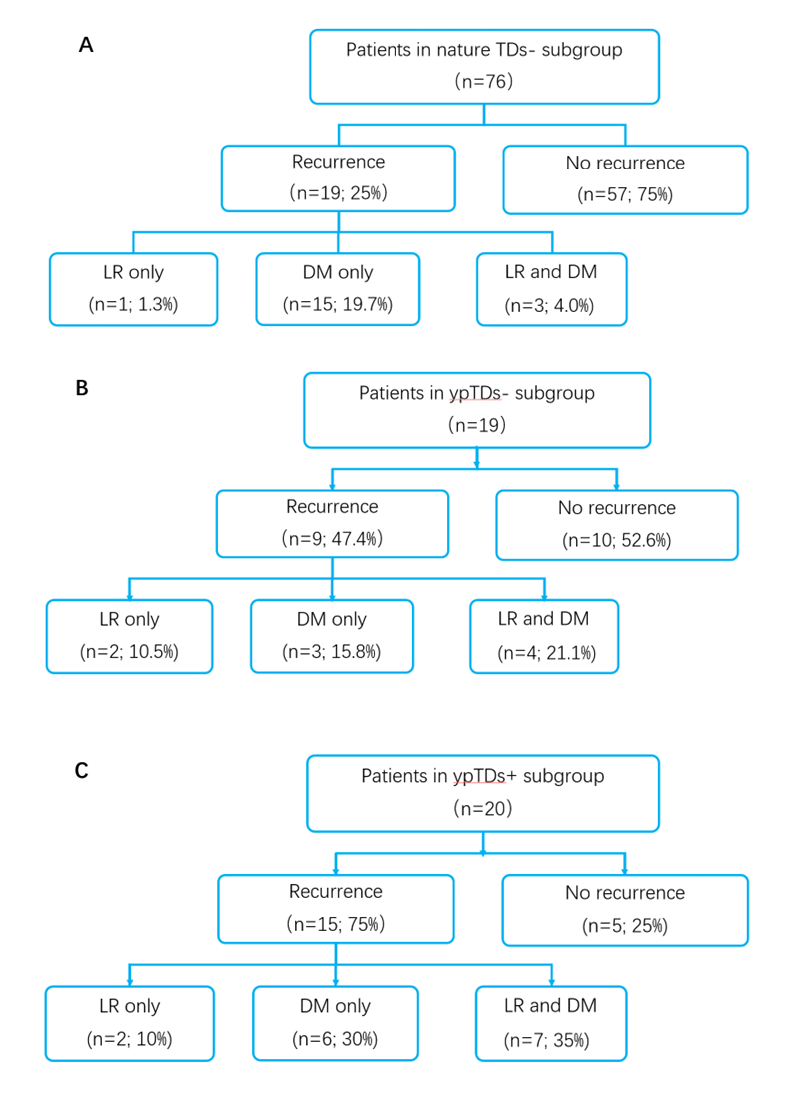

Supplement: S2 Fig — (TIFF) [file pone.0340000.s002.tiff]
