# Supplementary material for: Prognostic value of tumor deposits and their different response to neoadjuvant therapy in locally advanced rectal cancer
Source: PLoS One. 2026 Jan 13;21(1):e0340000. doi: 10.1371/journal.pone.0340000 (PMC12798964; doi:10.1371/journal.pone.0340000)
Supplement: S1 Table — (DOCX) [file pone.0340000.s003.docx]

**Supplementary1 table**

Table. Analysis of the relationship between mrTDs and ypTDs (n=115)

|  | ypTDs | |
| --- | --- | --- |
|  | - | + |
| mrTDs - | 76 | 2 |
| + | 19 | 18 |
| Total | 95 | 20 |
